# Mass Spectrometry-Based Proteomics Workflows in Cancer Research: The Relevance of Choosing the Right Steps

**DOI:** 10.3390/cancers15020555

**Published:** 2023-01-16

**Authors:** Paula Carrillo-Rodriguez, Frode Selheim, Maria Hernandez-Valladares

**Affiliations:** 1 Proteomics Unit of University of Bergen (PROBE), University of Bergen, Jonas Lies vei 91, 5009 Bergen, Norway; 2 Vall d’Hebron Institute of Oncology (VHIO), 08035 Barcelona, Spain; 3 Department of Physical Chemistry, University of Granada, Avenida de la Fuente Nueva S/N, 18071 Granada, Spain; 4 Instituto de Investigación Biosanitaria ibs.GRANADA, 18012 Granada, Spain

**Keywords:** mass spectrometry, proteomics, sample preparation, data-dependent acquisition (DDA), data-independent acquisition (DIA), workflows, data analysis, bioinformatics

## Abstract

**Simple Summary:**

Liquid chromatography–mass spectrometry (LC-MS)-based proteomics is a powerful technology for discovering new cancer biomarkers. In addition to last generation instrumentation, it uses experimental designs of different complexity that describe key steps from sample selection to data analysis and interpretation. All aspects must be optimized to obtain the most satisfactory results. However, planning proteomics procedures can be challenging unless their advantages and drawbacks are known. This review aims to highlight the methodological features that cancer researchers must consider before executing an LC-MS-based proteomics project. Based on these features, we suggest straightforward and complex workflows whereby researchers can discover new molecules or therapeutic pathways to defeat or significantly decrease the impact of oncological diseases.

**Abstract:**

The qualitative and quantitative evaluation of proteome changes that condition cancer development can be achieved with liquid chromatography–mass spectrometry (LC-MS). LC-MS-based proteomics strategies are carried out according to predesigned workflows that comprise several steps such as sample selection, sample processing including labeling, MS acquisition methods, statistical treatment, and bioinformatics to understand the biological meaning of the findings and set predictive classifiers. As the choice of best options might not be straightforward, we herein review and assess past and current proteomics approaches for the discovery of new cancer biomarkers. Moreover, we review major bioinformatics tools for interpreting and visualizing proteomics results and suggest the most popular machine learning techniques for the selection of predictive biomarkers. Finally, we consider the approximation of proteomics strategies for clinical diagnosis and prognosis by discussing current barriers and proposals to circumvent them.

## 1. Introduction

Liquid chromatography–mass spectrometry (LC-MS) proteomics is the current technology of choice for describing and quantifying the proteome of cells (as well as a single cell or subcellular fractions of cells), tissue, plasma, or other biological fluids and exosomes to understand the gene and cellular functions of particular conditions. Protein functions are usually identified by studying protein expression regulation, their posttranslational modifications (PTMs), and their protein–protein interaction networks. Thus, LC-MS-based proteomics analyses can provide a comprehensive picture of intra- and extra-cellular signaling [1].

Several LC-MS-based proteomics strategies are widely used to study the proteome of biological systems in medical research. They include top-down proteomics or the analysis of intact proteins (e.g., KRAS proteoforms in colorectal cancer cases); targeted proteomics used to verify, validate, and absolutely quantify candidate cancer biomarkers; and bottom-up or shotgun proteomics used to study whole proteomes [2,3,4,5,6,7]. The latter approach is widely utilized in the study of patient cohorts suffering cancer and other diseases. Shotgun proteomics workflows comprise several steps: selection of sample type, assessment of sample size, sample processing, data acquisition from the mass spectrometer, data cleaning and statistics, data interpretation and visualization, and machine learning. Unlike other omics technologies, there is little protocol standardization in LC-MS-based proteomics workflows, and therefore each project is carefully carried out according to a previously discussed experimental design of varied complexity depending on the number of samples and their nature, quantification method, enrichment of PTMs, and bioinformatics analyses.

LC-MS-based proteomics has been increasingly used in the search for disease biomarkers in the last decade. This has been accompanied by the continuous upgrading of mass spectrometers and the development of faster and more sensitive acquisition methods. In the recent past the identification of more than 2500 unique peptides per LC gradient minute and the quantification of approximately 5000 proteins with 21 min LC gradients have been reported for a quadrupole-Orbitrap mass spectrometer equipped with a differential ion mobility device [8]. Herein, we highlight several recent successes in which LC-MS-based proteomics has enabled the discovery of a classifier of five proteins (WAP four-disulfide core domain protein 2, WFDC2; prothymosin alpha, PTMA; nectin-4, PVRL4; fibrinogen alpha chain, FIBA; and nectin-2, PVRL2) for distinguishing between benign and malignant ovarian tumors [9], and a panel of six proteins (alpha-1-antichymotrypsin, AACT; trombospondin-4, TSP4; malate dehydrogenase mitochondrial, MDHM; calreticulin, CALR; protein LEG1 homolog, LEG1; and alpha-2-HS-glycoprotein, AHSG) for the detection of gliomas [10]. However, more studies are required to improve many of the current diagnostic assays as well as for the discovery of new prognostic biomarkers that will help us understand disease development and patient responses to treatments [11].

In cancer research, oncologists are aware of how LC-MS-based proteomics can significantly contribute to preclinical drug discovery. Nonetheless, multi-steps workflows employed in LC-MS-based proteomics and the multiple methodological choices available for each of them may overwhelm researchers that have not tried this technology before. For this reason, we present and describe these workflow steps, suggesting straightforward and branched roadmaps leading to the discovery of cancer biomarkers. Finally, we will discuss how proteomics strategies can be part of clinical diagnosis and prognosis assays, contemplating current barriers and encouraging proposals to circumvent them.

## 2. LC-MS-Based Proteomics Strategies from Sample Selection to Data Acquisition in Cancer Research: Steps and Main Considerations

**Figure 1:**
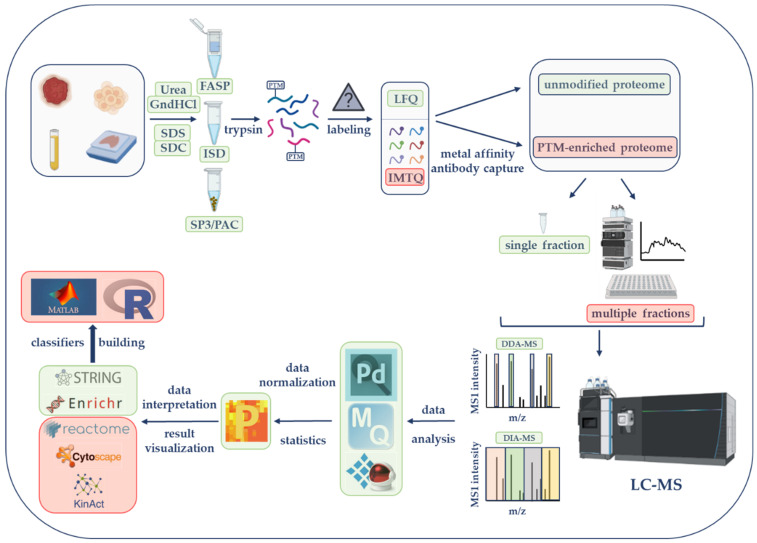
Liquid chromatography–mass spectrometry (LC-MS)-based proteomics workflow. Initial steps consist of sample lysis and solubilization in the presence of chaotropic agents (urea or guanidine hydrochloride, GndHCl) or detergents (e.g., sodium dodecyl sulfate, SDS; sodium deoxycholate, SDC). Samples are further processed by the filter-aided sample preparation (FASP), the in-solution digestion (ISD) procedure, single-pot, solid-phase-enhanced sample preparation (SP3), or protein aggregation capture (PAC) before trypsin digestion. According to the selected quantification approach, peptides will be kept unlabeled (for label-free quantification, LFQ) or will be labeled with tandem mass tags (TMTs) for isobaric mass tag quantification (IMTQ). A small portion will be utilized for the characterization of the unmodified or so-called global proteome while the rest of the sample will be used for posttranslational modifications (PTMs). Unmodified and modified samples can be analyzed as single fractions or as multiple fractions after being chromatographically fractionated. Peptides will be run on a mass spectrometer with data-dependent acquisition (DDA) or data-independent acquisition (DIA) methods. MS data will be analyzed by commercially or publicly available software followed by the utilization of several bioinformatics tools to perform gene ontology enrichment, protein network, and PTM characterization studies. These and more software and online applications are described in Table 1. The final step will involve the use of several artificial intelligence tools for classification modeling. The steps illustrated in this figure represent the most efficient strategies according to our experience. Steps framed with a green rectangle correspond to basic global proteomics workflows, while those framed with a light-red rectangle are utilized by experienced researchers or by those that seek PTM information. The figure was created with features obtained from BioRender (https://biorender.com/ (accessed on 25 May 2022)).

Each step of an LC-MS-based proteomics workflow represents an opportunity to maximize proteome coverage and obtain the most successful findings. Therefore, all possible approaches at each step must be carefully considered in order to create the most productive workflow. Here, we describe the core steps and propose simple and efficient tools to augment the quality and quantity of MS-based data (Figure 1). We will expand these workflows by adding steps covering PTM enrichment and machine learning for more experienced cancer researchers.

### 2.1. Sample Type’s Selection and Cohort Size

LC-MS-based proteomics can analyze any type of oncological samples from which proteins can be extracted. These include freshly frozen tissue or cells, formalin-fixed paraffin embedded (FFPE) tissue, blood fractions plasma or serum, feces, and other biological fluids such as urine, saliva, buccal swabs, and cerebral spinal fluid. While it might not be possible to select a sample’s type in some retrospective projects because of material availability, it is becoming easier to find different sample types (i.e., tissue and plasma) from the same patient thanks to the standard operating procedures (SOPs) that are being stablished in prospective studies by new biobanking policies [12,13]. In fact, the development and compliance of SOPs that include detailed criteria for proper sample collection (e.g., reagents and chemicals added; duration of the procedure) and storage (e.g., addition of cryoprotectants; storage temperature and acceptable duration) have become essential to guarantee sample quality and reduce variability of the project data. However, more efforts are required toward the elaboration of global SOPs that can facilitate sample sharing among different research groups and hospital biobanks.

Besides sample availability, three main factors determine the choice of sample type for proteomics research. The first factor is the tumor type and location. Biofluids in closer contact with tumors are probably a better source for potential biomarkers.

The second factor is the researcher’s skills along with equipment availability in the laboratory. The sample must be optimally processed in order to obtain the highest number of identified proteins and accurate quantitative values. Thus, while sample preparation for LC-MS analysis of leukemic blasts can involve uncomplicated procedures [14,15,16,17], FFPE tissue and plasma (key sample types in cancer proteomics) require more complex protocols that include reversal of chemical crosslinking, removal of reagents and protein extraction, and effective depletion of most abundant proteins, respectively [18,19,20]. Recently, a protocol that combines tissue disruption by ultrasonication, heat-induced antigen retrieval, and two alternative methods for efficient detergent removal has enabled satisfactory quantitative proteomic analysis of limited amounts of FFPE material [21]. Currently, plasma researchers are mainly using columns to selectively deplete the most abundant plasma proteins [22]. However, issues of reproducibility and indirect removal of relevant proteins have already been reported [23,24]. As an alternative, the use of nanoparticles with different surface chemistry was proven to identify ~4000 plasma proteins [25,26]. Nonetheless, the cost of this procedure, which is only available in a robotic system, becomes especially high in discovery studies.

The last factor to consider is the number of study subjects and their samples needed to achieve an acceptable study power, typically 80%. Although Levin demonstrated that for a study to be powered at 80% with a detectable fold change of 1.5 comparing two sample groups for all proteins, the minimum sample size was 60 per group [27], Nakayasu et al., found that the number of required biological replicates in a study of that power depends on the variability [11]. The variability in a study is the sum of the biological and the technical variability. Moreover, the study design (i.e., number of biological replicates and number of groups) depends on heterogeneity and homogeneity in a group or between groups. Therefore, it is important to identify samples that are homogenous in a group during the study design, and it is desirable that the groups to compare are as different as possible. Furthermore, the biological variability in a study is highly dependent on the sample origin; i.e., cancer cells are expected to have less variability compared to tumor tissue. The lower the variability in a study the higher the power of analysis, and as a result, a higher number of statistically changed proteins with smaller differences will be found.

The power of previously published LC-MS-based proteomics studies was rarely described. However, current journal practices and policies promote the inclusion of detailed descriptions of the experimental design that provide the necessary power of the study.

### 2.2. Sample Preparation Strategies

The choice of a sample preparation methodology is a key step of any LC-MS-based proteomics workflow [28,29]. Only the use of unbiased preparation approaches that produce a high number of identified and quantified proteins can provide satisfactory descriptions of the proteomes under study. Most sample processing for LC-MS analysis can be mainly categorized into in-solution (ISD), filter-based, and bead-based methods (Figure 1). While ISD protocols extract proteomes by the addition of concentrated solutions of chaotropic agents or detergents such as urea and guanidine hydrochloride (GndHCl) or sodium deoxycholate (SDC), respectively [30,31], filter-based or bead-based workflows allow protein extraction with detergents such as sodium dodecyl sulfate (SDS) or SDC and digestion in the presence of ammonium bicarbonate buffer after detergent removal. Other buffers such as HEPES and triethylammonium bicarbonate (TEAB) are used during digestion and are compatible with tandem mass tag (TMT)-labeling for relative quantification (see Section 2.3).

Although classical ISD protocols are less frequently used, recent attractive ISD solutions such as microreactor tips with on-column TMT labeling [32] and SDC-based ISD with TMT labeling in a 96-well plate format, SimPLIT [33], have been presented as efficient, fast, and low-cost approaches for the digestion of fluorescence-activated cell sorting (FACS)-sorted samples and global proteomics samples, respectively.

The first sample preparation and digestion methodology for MS-based proteomics using spin filters with a ≥3000 molecular weight cutoff membrane was introduced nearly two decades ago [34]. However, this method did not become popular in the proteomics community until it was presented as filter-aided sample preparation (FASP), which incorporated urea in a high concentration to successfully remove SDS [35]. Since then, FASP in combination with StageTip-based fractionation and multi-enzyme digestion FASP protocols has been extensively used for in-depth analysis of proteomes [36,37,38]. Magnetic bead-based sample preparation approaches for proteomics experiments were introduced, such as single-pot, solid-phase-enhanced sample preparation (SP3), and the protein aggregation capture (PAC). SP3 uses carboxylate-modified hydrophilic beads that bind proteins in a nonselective fashion through the use of ethanol-driven solvation capture. It is compatible with most of the common chemical agents used to facilitate cell or tissue lysis such as detergents, chaotropes, salts, and organic solvents [39]. As the entire SP3 procedure occurs in a single sample tube and takes little time when compared to other procedures [40], it is not surprising that the SP3 technology is becoming more and more popular among new and experienced MS-based proteomics researchers [41,42]. PAC, which employs the inherent instability of denatured proteins for non-specific immobilization on microparticles by aggregation capture, was shown to be more efficient than ISD and FASP procedures in the preparation of phosphopeptides and peptides from tissue and secretome samples [43]. Both protocols were also reported to be successful on automated devices [8,44,45,46].

To secure optimal sample preparation protocols for LC-MS-based proteomic studies aiming at the discovery of acute myeloid leukemia (AML) biomarkers, our research group has been testing novel techniques over the past few years. We started evaluating ISD and FASP proteomic workflows with leukemic blast samples isolated from peripheral blood [17]. Using two different quantitative approaches, label-free (LF) and stable isotopes labeling with amino acids in cell culture (SILAC), FASP workflows were selected to produce the highest number of quantified proteins with reduced number of missed cleavages. However, the use of fractionation methods such as the mixed mode with styrene-divinylbenzene-reverse phase sulfonate plugs in both FASP and ISD workflows, employing one (trypsin) and two proteases (Lys-C and trypsin) at the digestion step, respectively, quantified approximately 2200 proteins with an Orbitrap Elite mass spectrometer (Thermo Scientific, Waltham, MA, USA).

Because of the long processing time in the FASP procedure, we recently compared the performance of the ISD method using GndHCl in the lysis buffer and two proteases and the SP3 strategy using lysis buffers containing SDS or GndHCl and one protease with HeLa cell and human plasma samples [20]. Our results showed that the SP3 protocol, using either buffer, achieved the highest number of LF-quantified proteins in HeLa cells (5895–6131 without peptide fractionation; 7817–8136 with high pH reversed-phase LC fractionation) and plasma samples (397–411 without depletion and fractionation steps; 1397 after Top12 abundant protein depletion and high pH reversed-phase LC fractionation). Therefore, we have recently used the SP3 protocol with SDS-based lysis buffer for the proteomic analysis of AML samples [15,16].

Thus, we and other authors recommend the use of the SP3 procedure which represents a very robust and efficient processing tool for both concentrated and diluted protein materials [41,47,48,49]. To facilitate large studies, the use of automation (e.g., KingFisher^TM^ Flex, Thermo Fisher Scientific, Waltham, MA, USA) in a 96-well format was proved to have a great impact on the reproducibility of bead-based sample preparation protocols [8,50].

### 2.3. Quantification Strategies

Quantitative LC-MS-based proteomics experiments involve the use (or not, as in the LF quantification (LFQ) approach) of specific mass tags that are recognized by the instrument and are usually introduced into proteins or peptides metabolically or by chemical means, respectively [51]. SILAC utilizes the cell’s own metabolism to incorporate isotopically labeled amino acids into its proteome, which can be mixed with the proteome of unlabeled cells [52,53]. Thus, differences in protein expression can be analyzed by comparing the abundance of the labeled versus unlabeled proteins. The chemical derivatization processes include methodologies such as isotope-coded affinity tags (ICATs), dimethyl labeling, and isobaric mass tags among others [54,55,56,57,58]. Isobaric tags for relative and absolute quantification (iTRAQ), which consist of a reporter group, a balance group, and a peptide reactive group, are used to quantify up to eight peptide samples [59]. When the samples are pooled and analyzed simultaneously, the same peptide from the different samples will appear at the same mass in the MS1 scan. However, when the peptides are fragmented at the MS2 level, the peptide fragments provide amino acid sequence information and tag fragments, i.e., reporter ions. The ratios of these reporter ions are representative of the proportions of that peptide in each of the eight samples [59]. Herein, we will describe popular quantitative approaches with the use of TMT (another isobaric tag technique) and LFQ in current LC-MS-based proteomics.

LFQ was introduced early in the past decade as an alternative procedure to expensive and time-consuming stable isotope-based labeling methods. LFQ quantification is based on the intensities obtained from the extracted ion chromatogram (XIC) of MS1 signals or on spectral counting of the precursors, whereas peptide identification is carried out, as described for isobaric tag quantification, with peptidic features from fragment ions at MS2 [60]. It requires initial measurement of the sample concentration under consistent conditions and a strict adherence to the sample preparation workflow, including fractionation to resolve peptides with a consequent increase in the coverage of complex proteomes. LFQ has become highly employed in global proteomics and phosphoproteomics thanks to algorithms such as MaxLFQ, which handles fraction-dependent normalization information, calculation of pair-wise sample protein ratios from the peptide XIC ratios, and transfer of peptide identifications in one run to unidentified peptides in the subsequent run by matching their mass and retention times (i.e., the “match-between-runs, MBR” feature) [61]. Therefore, MBR can significantly increase the number of annotated identifications and provide more data for downstream quantification of proteins [62]. Recently, MBR has also been applied to TMT quantification using the three-dimensional MS1 features to transfer identifications from identified to unidentified MS2 spectra between LC-MS runs in order to utilize reporter ion intensities in unidentified spectra for quantification [63].

The TMT labeling system is used at the peptide level and consists of mass tagging reagents of the same nominal mass. Similar to ITRAQ labels, these tags are composed of an amine-reactive group, a spacer arm, and a mass reporter that are used for MS2 quantification. Commercial TMT kits (Thermo Fisher Scientific) contain 6, 10, 11, 16, or 18 labels (also called channels) that can be used in different experiment sets when a reference channel comprising a small aliquot from each sample serves as a normalization bridge among the different sets. This allows accurate quantification of large sample cohorts. Despite the tag cost, more and more proteomics researchers are using the TMT labeling approach since several optimized TMT labeling protocols covering important issues such as the peptide:tag ratio and reaction buffer have been recently published in addition to simplified commercial and free software workflows [64,65,66].

### 2.4. PTM Enrichments

The study of protein regulation by covalent modifications, PTMs, becomes necessary to understand the complexity and functionality of proteomes in cancer development [67]. PTMs that involve a mass increase in a peptide sequence can be identified and quantified with the LC-MS technology. Because of the substoichiometric abundance of many PTMs, their study involves enrichment procedures in order to remove unmodified peptides. The description and enrichment procedures of the most frequent PTMs are beyond the scope of this manuscript. However, we will herein focus on peptide phosphorylation as one of the major cellular signaling events, and we recommend several recent reviews regarding other PTMs and strategies to characterize them [1,68].

Phosphopeptide enrichment has been classically performed using metal oxide affinity chromatography (MOAC) with titanium dioxide beads, immobilized metal affinity chromatography (IMAC) with iron affinity gel, and sequential elution from IMAC (SIMAC) with a combination of both reagents [69,70,71]. Our group successfully constructed a dataset comprising more than 12,000 quantified class I (i.e., probability of site localization ≥ 0.75) phosphorylation sites from approximately 3000 proteins in an AML cohort with 41 patients using the IMAC protocol [72]. Nonetheless, the enrichment procedure has been remarkably eased by the use of magnetic material (e.g., MagReSyn Ti-IMAC HP beads from Resyn Biosciences) in the last few years [73].

### 2.5. Peptide Fractionation to Increase Proteome Coverage

Peptide fractionation is a necessary step before LC-MS analysis in order to achieve maximal proteome coverage in samples from complex organisms. Most popular fractionation techniques are based on peptide properties such as charge, polarity, and hydrophobicity [74]. Strong cation exchange, strong anion exchange, and mixed mode methodologies have been widely used as stuck disks on pipette tips or in the in-StageTip format [17,38,75]. However, in order to produce more fractions and take advantage of the increasing sensitivity of last generation mass spectrometers, offline high pH reversed-phase chromatography using C18 sorbents proved to be an excellent strategy to quantify up to 8434 mouse protein groups and 16,152 localized class I phosphosites when 46 and 12 TMT-labeled peptidic fractions were analyzed during a 30 min and a 60 min elution gradient, respectively [44]. Using the same number of peptidic fractions and length of LC gradients, 11,292 protein groups and 30,304 localized class I phosphosites were identified in HeLa lysates in an LFQ strategy [76].

Alternatively, high-resolution isoelectric point focusing (HiRIEF) applied at the (iTRAQ-labeled) peptide level in the 3.7–5.0 pH range identified 13,078 human and 10,637 mouse proteins when the 72 fractions obtained from the strip were analyzed during a 50 min gradient [77]. In a recent study, the analysis of TMT-labeled peptides from 141 non-small-cell lung cancer tumor samples that were fractionated on two strips (pH 3.7–4.9 and pH 3–10) and analyzed during a 60 min elution gradient quantified 13,975 proteins [78]. However, HiRIEF with two pH-range strips (2.5–3.7; 3–10) did not appear to efficiently perform in a cell-cycle arrest study that identified 19,075 localized class I phosphosites from a total of 132 TMT-labeled fractions analyzed during a 50 min elution gradient [79].

All things considered, the choice of peptide fractionation method is subject to the number of fractions that can be affordably analyzed, i.e., the MS time and the proteome depth sought.

### 2.6. MS Methods for Data Acquisition

LC-MS-based proteomics basically employs two MS data acquisition strategies, data-dependent acquisition (DDA) and data-independent acquisition (DIA), for global proteomics studies.

In DDA mode, the MS alternates between full-scan spectral acquisition at the MS1 level and MS2 sequential analysis of MS1 precursors selected according to their charge state (i.e., ≥2) and relative high intensity. Although this acquisition mode can be used for LF- or TMT-labeled samples, it introduces an abundance bias into the sampling and variability when running both biological and technical replicates. In order to alleviate these inherent DDA effects, the MS dynamic exclusion technology that adds masses with the highest intensity to a temporary exclusion list for a period of typically 30–60 s while peptides of lower abundance are sequenced and the already-mentioned software MBR tool have been widely used [61,80].

However, the development of new publicly available and commercial software solutions has encouraged the introduction and establishment of the DIA strategy in many proteomics platforms. In DIA mode, all MS1 precursors within a m/z range of interest are sequentially selected and fragmented at the MS2 level using isolation windows of different widths. It thus offers potentially deeper coverage of the data, decreasing the need for offline fractionation. As DIA does not suffer from the stochastic identifications of peptides that DDA suffers from, cross-sample comparisons in large cohorts are thus made much easier. Because of the complex deconvoluting processes of the fragmentation spectra, DIA is currently used for LF- and SILAC-spiked samples only. Originally, experimentally derived DDA run-spectral libraries were necessary to facilitate DIA spectral deconvolution. However, some current DIA applications that are discussed below (see Table 1) allow spectral analysis without their use.

Recent reports have shown that TMT–DDA methodology provides an excellent workflow to study proteomes and phosphoproteomes in depth. A TMT-based quantitative proteomic profiling of human monocyte-derived macrophages and foam cells identified 5146 proteins, among which 1515 and 182 were differentially expressed in macrophages/monocytes and foam cells/macrophages, respectively [81]. A three TMT 11-plex quantitative proteomic and phosphoproteomic analysis of human post-mortem cortex across asymptomatic phase Alzheimer’s disease, symptomatic Alzheimer’s disease, and healthy individuals identified 11,378 protein groups and 51,736 phosphopeptides [82]. However, DIA-based approaches that do not require expensive labels and time-consuming fractionation steps have become a powerful alternative for both proteomic and phosphoproteomics characterization [8,73,83]. A recent DIA with parallel accumulation-serial fragmentation (PASEF, a mass spectrometry technique that enables hundreds of MS/MS events per second at full sensitivity) study identified over 7700 proteins in HeLa cells in 44 min with quadruplicate single-shot injections and over 35,000 phosphosites after stimulation with epidermal growth factor in triplicate 31 min runs [84].

When TMT quantification is preferred, the synchronous precursor selection (SPS) MS3 technology in Orbitrap Tribrid mass spectrometers can be used to obtain a higher accuracy than the one provided by MS2 acquisition. Moreover, a real-time search (RTS) step between the MS2 and MS3 scans, which allows an MS3 scan acquisition only if the MS2 spectrum provides a positive peptide identification, can be selected in order to increase the scan rate of data acquisition and match the number of peptide identifications usually observed in MS2 acquisition [85,86,87].

## 3. LC-MS-Based Proteomics Data Analysis and Bioinformatics

Raw files with LC-MS data are complex and contain technical and biological information that must be properly visualized to interpret the data and communicate the results in a clear manner. We use visualization tools to examine the quality of the LC-MS data, to analyze the data at the peptide and protein level, and finally to show protein networks. Herein we describe our recommended LC-MS data and bioinformatics software and online tools that do not require any programming skills. However, R and Python scripts for data analysis and visualization are increasingly being used in the LC-MS-based proteomics community because of their capabilities, diversity, graphical quality, and the utility of their libraries. Therefore, programmer participation in proteomics projects is advantageous. Data analysis and visualization approaches with Python and R programming language can be found in other studies [88,89].

Initially in the data analysis workflow, LC-MS raw files can be analyzed with different software, based on the previous use of the FAIMS interface in the mass spectrometer. Presently, Proteome Discoverer (PD), PEAKS Xpro (Bioinformatics Solutions Inc., Ontario, Canada), and Spectronaut are the only software solutions that can fully handle ion mobility information from DDA and DIA datasets. However, DDA and DIA FAIMS-free LC-MS datasets can be analyzed with publicly available software such as MaxQuant [90,91], MSFragger [92], and DIA-NN [93] (Table 1). These software programs are supported by several online tutorials and workshops that can help beginners analyze their first LC-MS datasets (https://www.youtube.com/c/MaxQuantChannel (accessed on 8 August 2022); https://www.maxquant.org/summer_school/ (accessed on 12 August 2022); https://msfragger.nesvilab.org/; https://github.com/vdemichev/DiaNN (accessed on 22 August 2022)). DIA data analysis without the need for experimentally derived spectral libraries can be performed with DIA-NN, Spectronaut, and PEAKS Xpro.

There are several available platforms to clean and process LC-MS data. However, we recommend Perseus because of its diverse features including normalization, statistical testing, protein interaction, gene ontology (GO) enrichment, PTM analysis, machine learning, data visualization, and many more, thanks to an increasing number of plugins that are being continuously created by Perseus developers and others [94,95,96]. GO enrichment, pathways enrichment, and protein network analyses can be performed with tools other than the Perseus platform (Table 1). We recommend the sequential use of Enrichr-STRING-Cytoscape to investigate the effects of regulated proteins and visualize their interactions [97,98,99].

More experienced researchers can use the multiple LC-MS data management tools at OpenMS [100] and full analysis workflows at the Trans-Proteomics Pipeline (TPP; [101]). PTM studies using LC-MS-based phosphoproteomics can benefit from the use of WebLogo and IceLogo to create a graphical representation of the alignment of multiple amino acid sequences [102,103], KSEA and Kinact for kinase prediction [104,105,106], and Omnipath for kinase–substrate relationships [107].

**Table 1:** Recommended software and online applications * to analyze LC-MS-based proteomics data.

LC-MS Data	Data Processing	Gene Ontology (GO)/Pathway Analysis	Protein Networks	Visualization Tools
MaxQuant [90,91]	Perseus [94,95]	Enrichr [97]	STRING [98]	Cytoscape [99]
MSFragger [92]	Prostar [108]	A GO tool [109]	Omnipath [107]	OpenPIP [110]
DIA-NN [93]	Proteome Discoverer ^1^	Reactome [111]	PINA [112,113]	Perseus
Proteome Discoverer (F) ^1^	Qlucore ^1^	DAVID [114]	Perseus	
Mascot Distiller/Server ^1^		InnateDB [115]		
Spectronaut (F) ^1^		FunRich [116]		
PEAKS Xpro (F) ^1^ MSStats [117] Progenesis QI for Proteomics ^1^		QIAGEN IPA ^1^		

* Recommended software and applications are based on our own experience and the practice of renowned proteomics laboratories; ^1^ Software commercially available; F represents software that can analyze high-field asymmetric waveform ion mobility spectrometry (FAIMS) data.

## 4. Artificial Intelligence Strategies on Proteomics Data

Artificial intelligence (AI) is an area of computer science that can predict or classify objects or events driven by available data [118]. Through its algorithms, machine learning (ML), a discipline of AI, learns from data and makes predictions without being explicitly programmed.

LC-MS-based discovery proteomics studies that usually result in the identification and quantification of thousands of proteins must overcome the challenge of determining criteria or a strategy to prioritize biomarker candidates at the end of the workflow. Although statistical significance and fold change are the most frequent criteria when comparing groups, supervised ML is becoming recognized as a powerful approach to prioritizing biomarker candidates according to their performance in predicting the phenotype outcome [119,120]. LC-MS-based proteomics researchers are learning ML techniques such as logistic regression (LR), random forest (RF), K-Nearest Neighbors (KNN), and support vector machine (SVM). LR is a technique borrowed by ML from statistics, and it is used to predict the probability of a binary event occurring (e.g., acquiring ovarian cancer or not; sensitive or resistant to treatment). It has successfully associated proteomic subtypes with the risk of gastric lesion progression and identified salivary proteomic biomarkers for oral cancer screening [121,122]. RF models comprise multiple decision trees. A decision tree algorithm (typically the classification and regression tree, CART) is characterized by decision tree nodes to question the data until the leaf node is reached and the best split to subset the data is achieved. This modeling strategy has been successfully applied in the detection of biomarkers for prostate cancer progression and prediction of lung cancer from control cases and other tumors [123,124]. KNN is one of the simplest ML algorithms. It stores all the available data and classifies a new incoming data point based on the similarity with a well-suited category. KNN algorithms in combination with resampling and feature dimensionality reduction methods have recently contributed to cancer prediction using entropy data and improved the classification performance of lung cancer subtypes [125,126]. SVM aims to find a hyperplane that has the maximum margin (i.e., the maximum distance between data points of both classes) and distinctly classifies the data points. SVM works with kernel functions in order to determine the shape of the hyperplane and decision boundaries. This ML approach has been employed in the differentiation of breast cancer subtypes and for early detection of ovarian cancer [127,128]. These ML strategies can be run on the Perseus platform, in R or Python languages or with commercially available software such as MATLAB (MathWorks, Natick, MA, USA), Qlucore (New York, NY, USA), or JMP (SAS, Cary, NC, USA).

However, biomarker prioritization is not the only area of the LC-MS-based proteomics field where AI offers significant benefits. Despite the ongoing development of faster and more sensitive mass spectrometers by several companies, proteomics data never reach the completeness achieved by sequencing-based methodologies [129]. Therefore, it is expected that the AI contributions to several steps of the LC-MS-based proteomics workflow can substantially improve data quality and data interpretation. The impact of AI strategies on proteomics processes and data integration has been described in other publications [129,130]. In this section, we describe current approaches for MS2 prediction and peptide identification. To identify peptides at MS2, experimentally measured peptides are matched against ones calculated in silico using a sequence-reversed database to control the FDR. A widely used ML algorithm at this stage is the one of Percolator’s (available from PD software), which optimizes the number of true matches at a specified FDR by working with multiple peptide sequence features and experimental peptide data [131]. Another great ML tool is MS2PIP, which has achieved excellent correlations (0.9–0.95 Pearson correlation coefficient) between experimental and predicted spectra on different project datasets by considering the chemical properties of amino acids for spectral prediction [132,133].

Nonetheless, given the great number of peptides that can be obtained, especially from complex proteomes and of MS2 spectra available in public repositories such as PRIDE, Peptide Atlas, MassIVE, JPOST, iPROX, and Panorama within the ProteomeXchange Consortium (http://www.proteomexchange.org/ (accessed on 1 September 2022)) [134,135,136,137,138,139,140], the deep learning (DL) methodology is becoming more and more popular for MS2 prediction in our research community. As the fragmentation of a peptide bond depends not only on the adjacent amino acids but also on those far away from the bond, deep neural networks represent an ideal strategy to map the long-term sequential dependencies [129]. Popular neural-network-based DL software includes Prosit, whose models include different collision energies for peptide fragmentation [141], and DeepMass:Prism, which additionally takes into account the fragmentation energy and works with DDA and DIA data [142].

On the other hand, DL models are successful at detecting LC-MS features, at assessing spectral quality for identification, and at predicting the likeliest peptides and their corresponding proteins [129,130,143,144,145,146]. Moreover, DL strategies are becoming useful in the de novo peptide sequencing approach that aims to determine amino acid sequences using fragmentation data and without prior database knowledge. DeepNovo software is based on “automatically generating a description for an image”, where “image” represents MS2 spectra with intensity and mass/charge data. While convolutional neural networks (CNNs) are used to encode the “image”, long short-term memory (LSTM) recurrent neural networks (RNNs) are employed to describe the content of the “image” acquired in the DDA and DIA mode [147,148,149,150,151]. Additionally, DL algorithms are used to build classifiers that discriminate decoys and targets during the peptide identification process with DIA data (e.g., DIA-NN; [93]) and for the intensity-based rescoring of Sequest HT search engine results with the INFERYS workflow on the commercial PD platform [152]. At the last stage of the MS data analysis, CNN models can be run in protein inference strategies such as DeepPep, which quantifies the change in probabilistic score of peptide-spectrum matches (PSMs) in the presence or absence of a specific protein, hence selecting candidate proteins with the largest impact on the peptide profile [153].

Regarding the expertise requirements for including AI techniques in proteomics workflows, while ML approaches can be used by experienced proteomics researchers, the incorporation of DL algorithms requires a close collaboration with AI experts. This reinforces the complexity of MS-created data and the necessity to encourage the greater involvement of bioinformaticians and biomathematicians in LC-MS-based proteomics projects.

## 5. Fever of Single-Cell LC-MS-Based Proteomics

The study of the proteome of single cells has become an attractive solution to understanding the molecular basis of cell-specific functions and how cell types might respond to different stimuli. However, single-cell LC-MS-based proteomics must face several challenges associated with the low amount of protein material found in single cells (~150 pg) and with the proteome dynamic range expanding over several orders of magnitude [76,154]. It is indeed a technological scenario that requires much more expertise than that needed to perform bulk proteomics analyses.

There are several robotized and miniaturized sample preparation strategies that have been applied to the small-cell population or single-cell level. These include nanodroplet processing in one pot for trace samples (nanoPOTS) of glass chips with hydrophilic pedestals surrounded by a hydrophobic surface to serve as nanodroplet reaction vessels [155], nanoliter-scale oil-air-droplet (OAD) chips consisting of a stationary nanoliter microreactor with an oil-air-droplet sandwich structure [156], and commercial solutions such as cellenONE and proteoCHIP equipment (Cellenion, Lyon, France) that provide single cell sorting, isolation, and nanoliter acoustic dispensing for further sample processing including TMT labeling [157]. The latter approach identified approximately 2000 protein groups across 158 multiplexed single-cell samples from HeLa and HEK293T cultures. However, quantification of single-cell proteomes using the TMT approach was complicated by isotope cross talk between single-cell sample channels and the carrier channel, which contains peptides from one to several hundred cells and is employed to reduce the assay sensitivity from 10- to 200-fold [158,159]. Therefore, although MS sensitivity has significantly improved of late, allowing the use of a 25-cell sample in the carrier channel [160], LF procedures, and alternative methods for data acquisition such as DIA, which has fundamentally higher data completeness than DDA, are becoming more and more prevalent. The combination of PASEF and DIA has recently led to the quantification of up to 2000 proteins per single HeLa cell in a cell-cycle arrest experiment [161].

The proteomics strategies using isolated single cells described above become less attractive when tissues are under study. Procedures that analyze isolated cells from tissues will miss data regarding cell context, which is essential to fully understand cellular functions, cell-to-cell interactions (especially those between normal and cancer cells), and tissue heterogeneity. In order to retain their natural neighborhood, cells have been isolated by automated laser microdissection within a spatial region using a new approach called deep visual proteomics (DVP), which combines AI-driven image-based segmentation and classification for the analysis of cells showing a specific antibody-based bioimage [162,163].

## 6. Conclusions and Prospects

In cancer research, oncologists are aware of how LC-MS-based proteomics can significantly contribute to preclinical drug discovery. Nonetheless, multi-step workflows employed in LC-MS-based proteomics and the multiple methodological choices available for each of them might be overwhelming for researchers new to this technology. We here recommend the use of bead-based sample preparation followed by DIA with LFQ methodologies or DDA with TMT quantification for broad proteome and phosphoproteome characterizations. LC-MS-based applications in the clinic require fast and robust workflows. Therefore, bead-based sample preparation, phosphopeptide enrichment by the Ti-IMAC HP technique, and DIA represent an attractive protocol package for the characterization of cancer samples. These workflows have recently been applied to the analysis of different types of cancer samples providing, in some examples, 7000 phosphosites from FFPE lung biopsies with limited tissue amounts and 2103 proteins from plasma samples of breast cancer patients [164,165].

The study of patient samples from large cancer cohorts will benefit from the growing availability of efficient automated sample preparation methods that also enable PTM enrichment and improve the reproducibility of the data. Moreover, the introduction of new, robust, and fast chromatographic technology (Evosep, Odense, Denmark) greatly contributes to the feasibility of large cancer studies. Although sample preparation and data acquisition could be sequentially implemented in hospital laboratories, we must still put more effort into the development and engagement of bioinformatics solutions that provide a fast and easy interpretation of the proteomic data. Proteomics pipelines based on efficient workflows will be crucial in precision medicine decisions.
